# Comparative analysis of microbial communities from different full-scale haloalkaline biodesulfurization systems

**DOI:** 10.1007/s00253-022-11771-y

**Published:** 2022-02-11

**Authors:** Suyash Gupta, Caroline M. Plugge, Johannes B. M. Klok, Gerard Muyzer

**Affiliations:** 1 Wetsus, European Centre of Excellence for Sustainable Water Technology, Leeuwarden, The Netherlands; 2 Microbial Systems Ecology, Department of Freshwater and Marine Ecology, Institute for Biodiversity and Ecosystem Dynamics, University of Amsterdam, Amsterdam, The Netherlands; 3 Laboratory of Microbiology, Wageningen University & Research, Wageningen, The Netherlands; 4 Paqell B.V, Utrecht, The Netherlands

**Keywords:** Core community, Full-scale biodesulfurization processes, Haloalkaliphilic bacteria, Sulfide oxidation, Sulfide-oxidizing bacteria (SOB), *Thioalkalivibrio*

## Abstract

**Abstract:**

In biodesulfurization (BD) at haloalkaline and dO_2_-limited conditions, sulfide-oxidizing bacteria (SOB) effectively convert sulfide into elemental sulfur that can be used in agriculture as a fertilizer and fungicide. Here we show which bacteria are present in this biotechnological process. 16S rRNA gene amplicon sequencing of biomass from ten reactors sampled in 2018 indicated the presence of 444 bacterial Amplicon Sequence Variants (ASVs). A core microbiome represented by 30 ASVs was found in all ten reactors, with *Thioalkalivibrio sulfidiphilus* as the most dominant species. The majority of these ASVs are phylogenetically related to bacteria previously identified in haloalkaline BD processes and in natural haloalkaline ecosystems. The source and composition of the feed gas had a great impact on the microbial community composition followed by alkalinity, sulfate, and thiosulfate concentrations. The halophilic SOB of the genus *Guyparkeria* (formerly known as *Halothiobacillus*) and heterotrophic SOB of the genus *Halomonas* were identified as potential indicator organisms of sulfate and thiosulfate accumulation in the BD process.

**Key points:**

*• Biodesulfurization (BD) reactors share a core microbiome*

*• The source and composition of the feed gas affects the microbial composition in the BD reactors*

*• Guyparkeria and Halomonas indicate high concentrations of sulfate and thiosulfate in the BD process*

**Supplementary Information:**

The online version contains supplementary material available at 10.1007/s00253-022-11771-y.

## Introduction

Hydrogen sulfide (H_2_S) is a malodorous, toxic, and corrosive gas, which can be harmful to all forms of life. This gas is produced in nature by sulfate-reducing bacteria found in anaerobic habitats, such as marine and freshwater sediments, as well as man-made processes (Muyzer and Stams 2008). Naturally, geothermic activity also releases H_2_S in the atmosphere. Nowadays, the petrochemical industry is the largest anthropogenic producer of SO_2_, produced upon combustion of H_2_S. The other sources include paper mills, the textile industry, biogas plants, landfills, and the rendering industry (Mahmood et al. 2007; Janssen et al. 2009). Conventional methods to remove H_2_S on-site include physical and chemical processes. Alternatively, more environmentally friendly and cost-effective haloalkaline BD processes employ haloalkaliphilic SOB to convert sulfide into elemental sulfur at haloalkaline conditions and low redox potential (Janssen et al. 2001). In these processes, H_2_S is first absorbed into an alkaline solution as a predominantly soluble bisulfide (HS^−^). The HS^−^-loaded solution is subsequently directed to a bioreactor whereby SOB oxidizes it to elemental sulfur at a low redox potential of around −300 mV (Sorokin et al. 2008; Janssen et al. 2009). The produced sulfur is harvested from a settler and can be further reused as fertilizer or fungicide. With this BD process, 99.84% of sulfide can be removed (de Rink et al. 2020). Besides sulfur, thiosulfate and sulfate are formed as side products through chemical oxidation of polysulfide and complete oxidation of reduced sulfur compounds mediated by SOB, respectively. Both compounds are undesirable, as their formation (i) increases acidification and consequently, the need for extra caustic supply, (ii) increases the demand of oxygen supply (= energy consumption), and (iii) decreases the sulfur selectivity of the process (van den Bosch et al. 2007). The rate of formation and removal of sulfate and thiosulfate from the process determines the concentration of these compounds in the reactor system and can be regarded as indicators of a less efficient process. We hypothesize that these indicators are directly linked to the activity of particular bacteria in the reactor.

Aerobic sulfur oxidation is largely performed by chemolithoautotrophic SOB along with facultative autotrophs, lithoheterotrophs, and obligate heterotrophs. The chemolithoautotrophic SOB possesses the ability to conserve energy from the oxidation of reduced sulfur compounds, such as sulfide, polysulfides, sulfur, thiosulfate, and polythionates with oxygen or nitrate as electron acceptor (Muyzer et al. 2013). They are members of four genera of haloalkaliphilic *Gammaproteobacteria*: *Thioalkalimicrobium* reclassified as *Thiomicrospira* (Boden et al. 2017), *Thioalkalispira, Thioalkalivibrio*, and *Thioalkalibacter* (Sorokin et al. 2013, 2015). The haloalkaliphilic *Thiomicrospira* and *Thioalkalispira* species can grow with thiosulfate or sulfide forming mostly sulfate as a product but can produce elemental sulfur under oxygen-limiting conditions (Sorokin et al. 2001; 2002). The genus *Thioalkalivibrio* includes multiple isolates (more than a hundred) from natural soda lakes and haloalkaline bioreactors that can oxidize various reduced sulfur compounds to sulfur or sulfate using oxygen or nitrate as an electron acceptor (Berben et al. 2019). Until today, ten species of *Thioalkalivibrio* have been well-characterized and a variety of other species have been identified based on their genomes (Ahn et al. 2017; Berben et al. 2019). Other SOBs from haloalkaline habitats are members belonging to the *Alkalispirillum–Alkalilimnicola* group, i.e., *Alkalilimnicola halodurans, Alkalilimnicola ehrlichii* MLHE-1 (Hoeft et al. 2007), *Alkalispirillum mobile* (Rijkenberg et al. 2001), and several unclassified members. Members of the *Alkalispirillum–Alkalilimnicola* group are facultatively autotrophic genera that can utilize CO as an additional *e*-donor (Sorokin et al. 2006)*.* Other bacteria that might be present in the haloalkaline bioreactors are members of the genus *Halomonas*, which are heterotrophic bacteria that can oxidize thiosulfate to tetrathionate, which, in turn, can chemically oxidize sulfide to sulfur with the regeneration of thiosulfate, thus, potentially contributing to the overall sulfide oxidation to sulfur (Sorokin, 2003; Sorokin et al. 2008).

Multiple 16S rRNA sequence analyses of bacterial communities from some full- and pilot-scale BD processes has revealed the presence of both chemolithoautotrophic and heterotrophic SOB belonging to the *Gammaproteobacteria*, including *Thioalkalivibrio*, *Thiomicrospira*, and *Halomonas* species (de Graaff et al. 2011; Sorokin et al. 2012; Roman et al., 2016; Kiragosyan et al. 2019). Recently, the BD process has been improved by the addition of an anoxic reactor in series (Klok et al. 2017, 2018). In this BD concept, SOB was exposed to alternating conditions of subsequent anaerobic-sulfidic and microaerobic conditions. In the new process, line-up selectivity for sulfur formation of 96.5% was achieved, which considerably decreases NaOH consumption and bleed stream formation compared to the line-up without the anoxic reactor. Correspondingly the dominance of the facultative chemolithoautotrophic SOB *Alkalilimnicola* also increased in the novel BD process (de Rink et al. 2019). Although the aforementioned studies provide insight into the microbial community composition of some BD processes, detailed comparative information on the microbial community composition in different full-scale plants is still lacking. Besides, the presence of a possible core community in the BD process has not been studied before.

In this paper, we analyzed the microbial communities from eight full- and two pilot-scale biodesulfurization reactors along with a variety of physicochemical parameters. We determined the microbial diversity in these bioreactors and defined the presence of a core community. Moreover, we revealed a relation between the presence of specific community members at particular environmental conditions, such as alkalinity. We also showed that there are certain bacteria that can be linked to an increase in the undesired production of thiosulfate and sulfate.

## Materials and methods

### Description and sampling of full-and pilot-scale BD reactors

To determine the microbial community composition of BD reactors comprehensively, process solutions of eight full-scale BD reactors and two runs of a pilot-scale BD reactor were analyzed in 2018. Full-scale BD reactors studied were from industries that treated feed gas from different origins (Table 1). The pilot-scale BD reactors consisted of an extra reactor, namely an anoxic reactor in front of the oxic reactor. The difference between the pilot-scale and full-scale was the composition of the feed gas; for the pilot scale BD reactors, a synthetic gas with no contaminants was used, while the full-scale plants have organic sulfur compounds and hydrocarbons in the feed (Table 1). The process solution was collected from full-scale bioreactors sampling points. The solution was used to analyze the physical-chemical parameters and to extract genomic DNA for amplicon sequencing. For the pilot-scale BD reactors, the samples were collected from the sludge preserved at 4 °C after the process operation.

**Table 1:** Description of biodesulfurization plants

*Reactor name*	*Denotation*	*Plant description*					*Physicochemical parameters*							
	*Sample ID (replicate IDs)*	*Industry*	*Feed gas type*	*Location*	*Sulfur separation*	*Additional components in the feed gas*	*ORP at source (mV)*	*H*_*2*_*S/CO*_*2*_	*pH*	*Conductivity (mS/cm)*	*Salinity (Na*^*+*^*, M)*	*Alkalinity (M) HCO*_*3*_^*-*^	*Sulfate (M)*	*Thiosulfate (M)*
Cadaver	Cad1 (a. b, c)	Rendering	Biogas	Son, NL	Effluent settler	Ammonia	-347*	0.04	8.67	78.64*	1.55*	0.18*	0.53*	0.16*
Landfill	LF1 (a. b, c)	Landfill waste	Landfill gas	Amersfoort, NL	Effluent settler	None	-283	0.01	8.40	40.68	0.90	0.71	0.06	0
Pilotunit_01	Pil1 (a. b, c)	None	Synthetic gas	Wageningen, NL	Decanter centrifuge	None	-370**	0.02	8.14	49.49	1.02	0.42	0.29	0.02
Pilotunit_02	Pil2 (a. b, c)	None	Synthetic gas	Wageningen, NL	Decanter centrifuge	Oxygen	-370**	0.02	8.59	40.14	0.81	0.55	0.14	0.02
Papermill_01	PM1 (a. b, c)	Papermill	Biogas	Eerbeek, NL	Effluent settler	None	-389	0.06	8.77	64.08	1.48	0.40	0.38	0
Papermill_02	PM2 (a. b, c)	Papermill	Biogas	Roermond, NL	Effluent settler	None	-377*	0.06	8.80	44.07*	0.78*	0.55*	0.1*	0.02*
Papermill_03	PM3 (a. b, c)	Papermill	Biogas	Cuijk, NL	Effluent settler	None	-360	0.06	8.66	54.72	0.97	0.29	0.32	0
Papermill_04	PM4 (a. b, c)	Papermill	Biogas	Zulpich, DE	Effluent settler	None	NA	0.06	8.89	62.16	1.17	0.57	0.28	0.05
Oil&Gas_01	OG1 (a. b, c)	Oil and gas	Associated gas	Southern Illinois, USA	Decanter centrifuge	Thiols	NA	1.67	9.03	50.66	0.89	0.49	0.03	0.16
Oil&Gas_02	OG2 (a. b, c)	Oil and gas	Amine acid gas	Texas, USA	Decanter centrifuge	BTEX	NA	0.02	8.2^+^	85^+^	1.5	0.85^+^	0.36	0.03

*These measurements were taken from (Mol et al. 2020), + The value represents the average operational value, ** ORP set point of the BD reactor, NA These values are not available as the samples were not collected at the source.

### Physicochemical analyses of the bioreactor samples

To investigate the physical-chemical parameters of the collected samples, the samples were processed in the laboratory as early as possible. Prior to the analysis, the samples were kept at 4 °C and were filtered over a 0.45 µm membrane filter (HPF Millex, Merck, the Netherlands). Thiosulfate, sulfate, and sodium were measured by ion chromatography as described by Roman et al. (2015). Total inorganic carbon was quantified using a TOC-VCPH/CPN analyzer (Shimadzu, The Netherlands). Carbonate and bicarbonate ions concentration were estimated from the total inorganic carbon using the Henderson-Hasselbalch equation (Po and Senozan 2001) and further used to express the alkalinity of the system as concentration NaHCO_3_ (de Rink et al. 2019). The pH and conductivity of the well-mixed unfiltered samples were measured using the kit from Mettler Toledo, USA.

### DNA extraction and 16S rRNA gene amplicon sequencing

For the microbial community composition analysis, genomic DNA was extracted from samples collected from the BD reactors in triplicate from each bioreactor sample. Twenty milliliters of sludge was centrifuged at 10,000 × *g* for 10 min at room temperature. The supernatant was discarded, and the pellet was suspended in a buffer containing 1M NaCl and phosphate-buffered-saline (PBS) to maintain the osmotic balance of the cells. The biomass was centrifuged again, and the obtained mixture of biomass and sulfur was used for the DNA extraction. The DNA was extracted using DNeasy PowerLyzer PowerSoil Kit (Qiagen, Germany) following the manufacturer’s instructions. The DNA was purified with DNA Clean & Concentrator-5 kit (Zymogen, USA). The integrity of the extracted genomic DNA was visualized by gel electrophoresis. PCR with 16S rRNA gene primers 27F and 1492R was performed, and amplicons were visualized by gel electrophoresis to verify the relative intensity of the amplicons. The extracted DNA was quantified using the QuantiFluor dsDNA system on a Quantus fluorometer (Promega, USA). Samples were diluted to a final concentration of 30 ng/µL of DNA and sent for Illumina MiSeq sequencing performed at commercial sequencing company MR DNA (www.mrdnalab.com, Shallowater, TX, USA). At the facility, samples were first amplified in triplicate using primers 515f (5ʹ-GTGYCAGCMGCCGCGGTAA-3ʹ) and 926r (5ʹ-CCGYCAATTYMTTTRAGTTT-3ʹ) with a bar-coded forward primer. This primer set covers the 16S rRNA gene V4–V5 variable region (Parada et al. 2016). Amplification was done three times using the HotStarTaq Plus Master Mix Kit (Qiagen, USA) under the following conditions: 94 °C for 3 min, followed by 30 cycles of 94 °C for 30 s, 53 °C for 40 s, and 72 °C for 1 min, with a final elongation step at 72 °C for 5 min. After amplification, the PCR products of three separate reactions of each sample were pooled, and PCR products were checked on a 2% (*w/v*) agarose gel to determine the success of amplification and the relative intensity of the bands. Then, multiple samples were pooled together in equimolar amounts based on their molecular weight and DNA concentrations. Pooled samples were purified using calibrated Ampure XP beads (Beckman Coulter, Indianapolis, IN, USA) and used for sequencing on a MiSeq system following the manufacturer’s guidelines. The sequence data has been submitted to the ENA database under study number PRJEB43104.

### Amplicon sequence analysis

The 2 × 300 bp paired-end reads were generated by Illumina MiSeq sequencing. The reads generated were converted to paired-end format from a mixed paired format using the fastq processor software provided by MR DNA which removes the barcodes, primers and corrects the orientation of the reads. Microbiome analysis was performed with QIIME-2 version 2020.2 (Bolyen et al. 2019). Raw sequence data were demultiplexed and quality filtered using the q2‐demux plugin followed by denoising with DADA2 (Callahan et al. 2016) (via q2‐dada2) to remove the errors and chimeric sequences and identify all observed amplicon sequence variants (ASVs) (100% operational taxonomic units). All amplicon sequence variants (ASVs) were aligned with MAFFT (Katoh and Standley 2013) (via q2‐alignment) and used to construct a phylogeny tree with fasttree2 (Price et al. 2010) (via q2‐phylogeny). The taxonomy was assigned to the ASVs using the q2‐feature‐classifier (Bokulich et al. 2018), the classify-sklearn naive Bayes taxonomy classifier (Pedregosa et al. 2011) trained on nearly complete 16S rRNA sequences from the SILVA database version 138 (Quast et al. 2013; Yilmaz et al. 2014).

Alpha‐diversity metrics (observed OTUs and Shannon), beta diversity metrics (Bray‐Curtis dissimilarity), and Principal Coordinate Analysis (PCoA) were estimated using q2‐diversity after samples were rarefied (subsampled without replacement) to 33,340 sequences per sample.

### Identification and phylogenetic analysis of the core community members

The core ASVs were identified using the QIIME 2 feature table core features plugin. To create a visual diagram depicting the core community, the ASV table was filtered based on industry type to generate five ASV tables specific to each industry type using QIIME 2 feature table filter samples plugin. The core ASVs for each industry type were identified using the QIIME 2 feature table core features plugin. The counts of core ASVs were used to create a Venn diagram to show the shared core community of all the BD reactors. The Venn diagram was created using the “InteractiVenn” tool (Heberle et al. 2015) whereby, each industry type is represented as one set. Next, the core ASV table for the whole data set was created using QIIME 2 feature table core features plugin. The representative sequences were filtered based on the core ASV tables using QIIME 2 filter sequences tool. The identified core sequences were aligned in SINA alignment (Pruesse et al. 2012) imported into ARB (Quast et al. 2013; Yilmaz et al. 2014) to create a phylogenetic tree showing the core ASVs and their close relatives.

### Prediction of the functional potential of the core microbiome

The functional potential of the core community was predicted using the software program Tax4Fun2 that can predict the functional capabilities of the microbial communities based on 16S rRNA datasets (Wemheuer et al. 2018). The genomes of bacteria that were closely affiliated to the core community members were also included in the analyses as described in the pipeline (https://github.com/bwemheu/Tax4Fun2).

### Co-occurrence network analysis

Co-occurrence networks were calculated with the software program GenePiper (Tong and Chan 2020) using the raw read counts as abundance type. In addition, we used the person correlation method; the correlation filter was set to 0.8 and the *P*-value to 0.05, and agglomeration was performed at the genus level.

### Statistical analysis of the data

To identify the difference in microbial community composition between samples and different industry types, PCoA analysis was performed using the Bray Curtis similarity distances. Redundancy Analysis (RDA) was performed to determine which parameters among pH, salinity, alkalinity, conductivity, H_2_S/CO_2_ ratio, and concentrations of thiosulfate and sulfate significantly explain variation in microbial community composition. The analysis was performed in an R software environment (version 3.6.3) using the vegan package. The ASV table, tree, and taxonomy files created in QIIME 2 were imported in R for this analysis. As a prerequisite to RDA, the species frequency is converted to abundance data. Variance inflation factors were analyzed for all parameters to confirm the non-collinearity using package car (version 3.0-8). The significance of explanatory variables was analyzed using ANOVA-like analysis with 999 permutations, and a tri-plot was generated using the ggplot2 package in R.

In addition to the relationship, we were also interested in the differential abundance of ASVs/taxa with changing concentrations of sulfate, thiosulfate, and alkalinity. The differentials were generated using the q2-songbird plugin in QIIME 2 (Morton et al. 2019) by fitting environmental parameters to the multinomial regression model. The good fit was determined using the factor *Q*^2^. A value closer to 1 indicates a good fit and therefore represents the significant differential abundance of species due to change in parameter values. A value closer to 0 indicates a poor fit and no conclusion can be inferred from the model.

## Results

### Physicochemical analyses

Several physicochemical parameters of the full-scale BD-processes, such as pH, salinity, conductivity, alkalinity, and concentrations of sulfate and thiosulfate, were determined and are presented in Table 1. The BD-reactor treating biogas from the rendering industry had the highest measured sulfate and thiosulfate concentrations and the lowest alkalinity. In contrast, the BD-reactor that was treating gas from a landfill had low sulfate and thiosulfate concentrations but high alkalinity. In the BD processes that were treating gas from the petrochemical industry, only sulfate was detected. For all samples, the salinity varied between 0.8 and 1 M Na^+^, and the conductivity was between 40 and 78 mS/cm. The alkalinity ranged from 0.18 to 0.71 M and the pH from 8.1 to 9, indicating haloalkaline conditions in all reactors. The oxygen supply in all plants was controlled using ORP (van den Bosch et al. 2007). The measured range of the ORP at the installation ranged from −389 to −283 mV.

### 16S rRNA gene-based amplicon sequencing and microbial diversity analyses

Demultiplexing and denoising of the raw Illumina MiSeq sequences from the 30 biomass samples of the BD-reactors resulted in 33,342 to 73,228 reads and a total of 444 ASVs were assigned (Supplementary Table S1). Alpha diversity, which is indicative of the mean species diversity, based on both observed ASVs and Shannon indices varied significantly among the different BD reactors (Supplementary Fig. S1, Kruskal–Wallis, *H* = 17.81, *p* = 0.03 for observed ASVs and *H* = 27.64, *p* = 0.001 for Shannon).

**Fig. 1:**
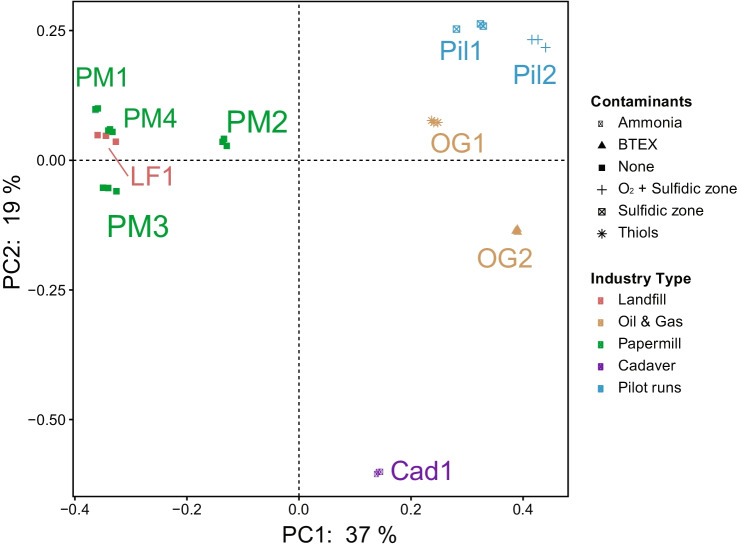
Principal coordinate analysis (PCoA) of Bray Curtis dissimilarity distances of the microbial communities of biodesulfurization reactors from different industries (see Table 1)

The beta diversity, indicative of the difference between microbial communities from different samples, was significantly different among the samples (Bray Curtis distance; PERMANOVA: *F* = 13.9778, *p* = 0.001, permutations = 999). The Bray Curtis dissimilarity distances based on PCoA analysis (Fig. 1) showed great similarity in the microbial community composition of reactor samples from the paper mill industry and landfill gas treating plants, while the community composition of plants from the rendering industry and the petrochemical industry were more distinct. The BD reactors with no contaminants such as hydrocarbons and organic sulfur compounds in the feed gas had a less diverse community as compared to the BD reactors with a larger organic matter input. This was also observed for the two pilot plants that had an additional anoxic reactor. Two BD reactors employed in Oil and Gas industries had different contaminants in their feed gas, such as BTEX and thiols (Table 1), and had a different microbial community composition. The BD reactor of the rendering industry had high ammonia content and a very different microbial community composition than the other BD-reactors. The H_2_S/CO_2_ ratio in the feed gas and sulfur separation method did not have much effect on the microbial diversity among different BD reactors (Supplementary Fig. S2).

### Identification of a core microbial community in the BD reactors

**Fig. 2:**
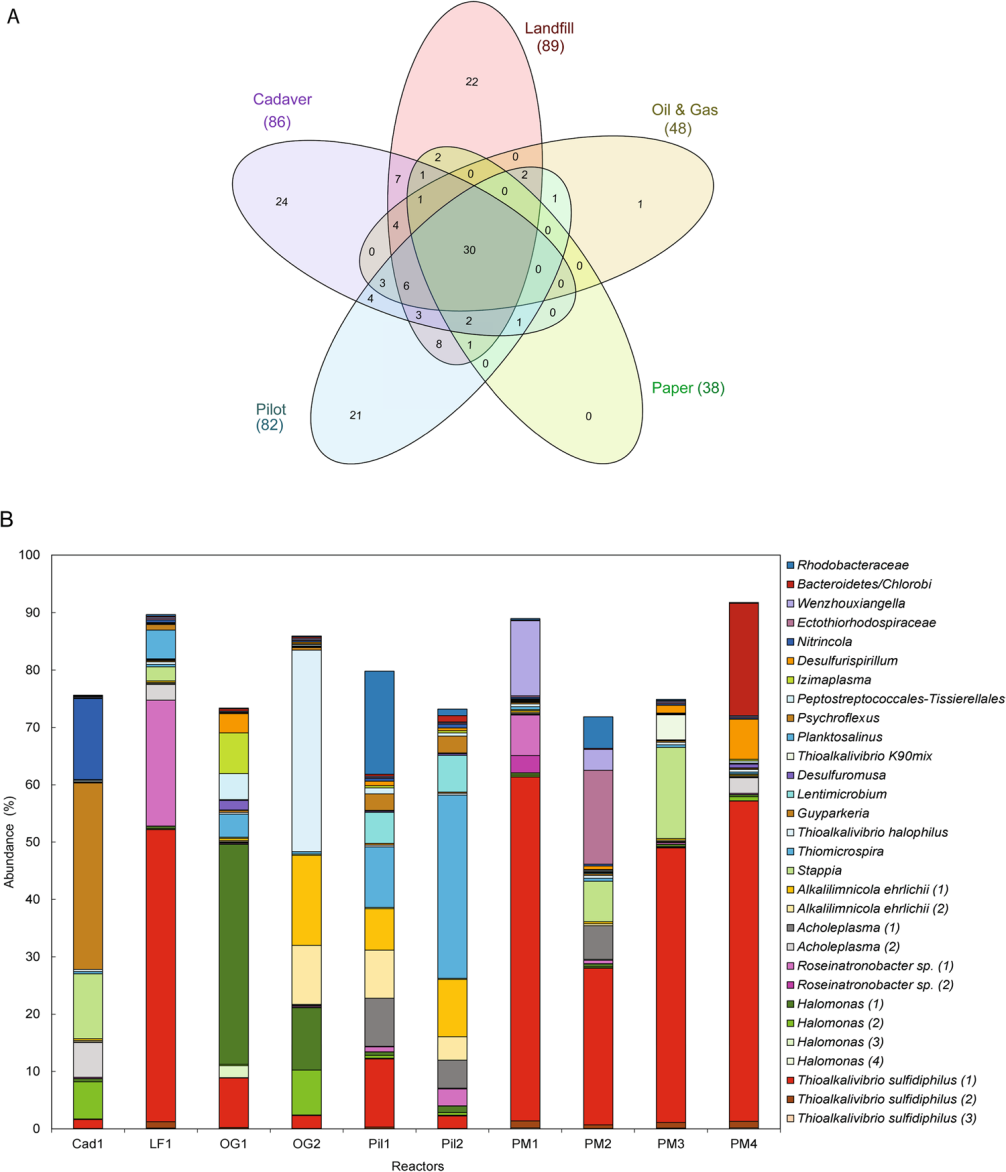
Core community members found in the biodesulfurization reactors. **A** Venn diagram showing the number of unique and common ASVs. Sets represent the industry type the plants treat. The total count of each set is the number of ASVs commonly occurring in the reactors treating that industrial effluent. **B** Bar graph showing the average relative abundance of the 30 core ASVs in three replicates of each sample

The core community of the bioreactor samples was defined based on the ASVs that were present in all BD-reactors. A total of 30 ASVs were present in all samples indicative of the “core microbial community.” The Venn diagram shown in Figure 2A indicates the distribution of all 444 ASVs among the samples grouped based on the industrial waste they treat and the 30 ASVs that are common to all BD-reactors studied. Interestingly these 30 ASVs together account for 72 to 90% of total microbial diversity in BD reactors (Fig. 2B).

### Taxonomy-based analyses

Taxa assignment to identify ASVs revealed that microbial community members from the class *Gammaproteobacteria* had the highest relative abundance. These included the genera *Thioalkalivibrio*, *Alkalilimnicola*, *Guyparkeria*, *Halomonas*, *Alkalispirillum*, *Vibrio*, and *Thiomicrospira*.

The core microbial community with 30 ASVs consisted of 16 members from the class *Gammaproteobacteria* with known SOBs *Thioalkalivibrio sulfidiphilus*, *Thioalkalivibrio halophilus*, *Thioalkalivibrio* sp. K90mix, *Alkalilimnicola ehrlichii*, *Halomonas*, *Thiomicrospira*, and *Guyparkeria* along with *Nitrincola* and *Wenzhouxiangella*. Four members belonged to the class *Alphaproteobacteria* that includes members of genera *Roseinatronobacter* (a known haloalkaliphilic lithoheterotrophic SOB), *Stappia*, and one unclassified member of the family *Rhodobacteraceae.* The other four belonged to class *Bacteroidia* that includes members of genera *Planktosalinus*, *Psychroflexus*, *Lentimicrobium*, and Blvii28_wastewater-sludge_group*.* The next three members belonged to class *Bacillia* which includes members of genera *Acholeplasma* and *Izimaplasma*. The latter was also present among the abundant metagenomes recovered recently from Siberian soda lakes (Vavourakis et al. 2019). The final three members of the core community belonged to the class *Clostridia*, class *Desulfuromonadia*, and class *Chrysiogenetes*, which includes *Peptostreptococcales*-*Tissierellales*, *Desulfuromusa*, and *Desulfurispirillum*, respectively. The relative abundance of each core community member is shown in Fig. 2B. The most abundant bacterium was *Thioalkalivibrio sulfidiphilus* with a relative abundance of up to 60%.

**Fig. 3:**
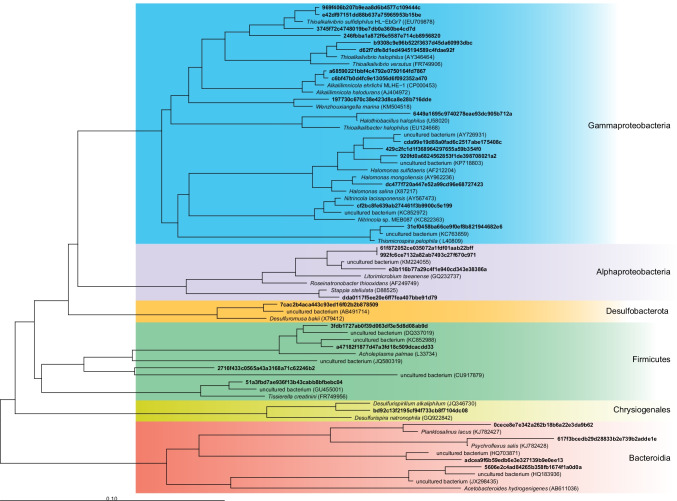
Phylogenetic tree showing the affiliation of bacterial populations represented by the sequences of the 30 ASVs to closely related cultured and uncultured bacteria

To visualize the phylogenetic affiliation of the core community members, a phylogenetic tree was created with the 30 ASVs with their cultured and uncultured close relatives (Fig. 3). The tree shows that the members of the core community are affiliated with various phyla.

### Prediction of the functional potential of the core community

**Fig. 4:**
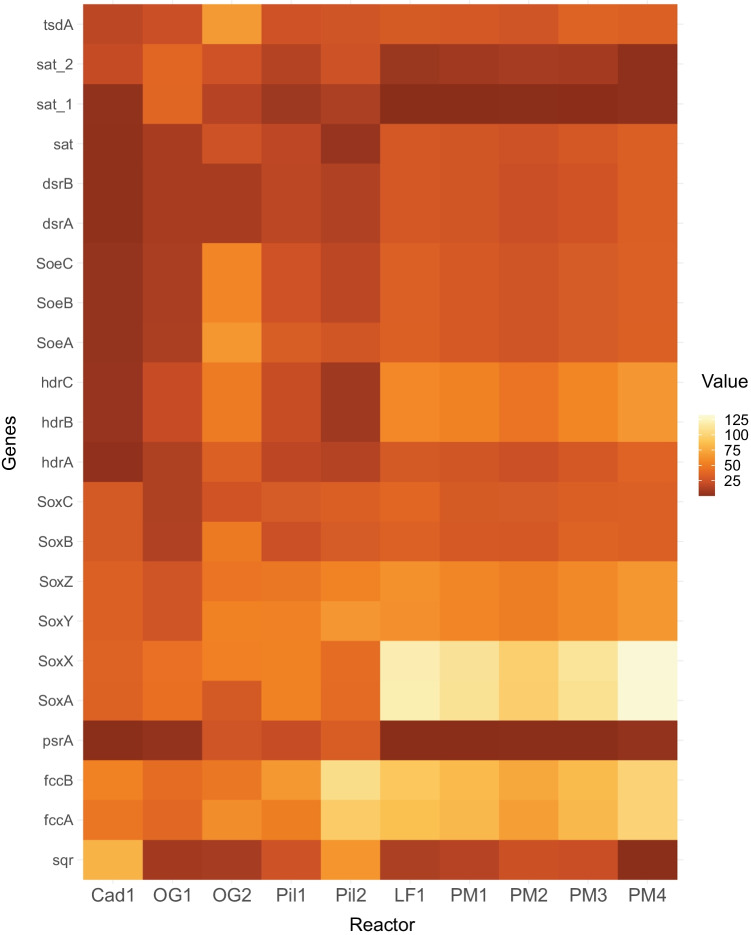
The relative abundance of predicted functional genes by Tax4Fun2: the raw values were multiplied by a factor of 10^5^ to create the input matrix for the heatmap

Using Tax4Fun2, we focused on the genes involved in the oxidation of reduced sulfur compounds. The predictions indicate the presence of all genes needed for complete sulfide oxidation to sulfur (Fig. 4; Table S2). The sulfide-oxidizing genes sqr, fccAB (Griesbeck et al. 2000; Trüper et al. 2002), and thiosulfate-oxidizing genes soxABXYZ were found to be highly abundant in the reactor samples from the paper mill (PM) and from the landfill (LF). We also searched for genes involved in the conversion of contaminants such as ammonia, thiols, and BTEX. Only BTEX degrading genes were found to be present in low numbers in oil and gas plants and pilots plant (Table S2).

### Co-occurrence analysis

**Fig. 5:**
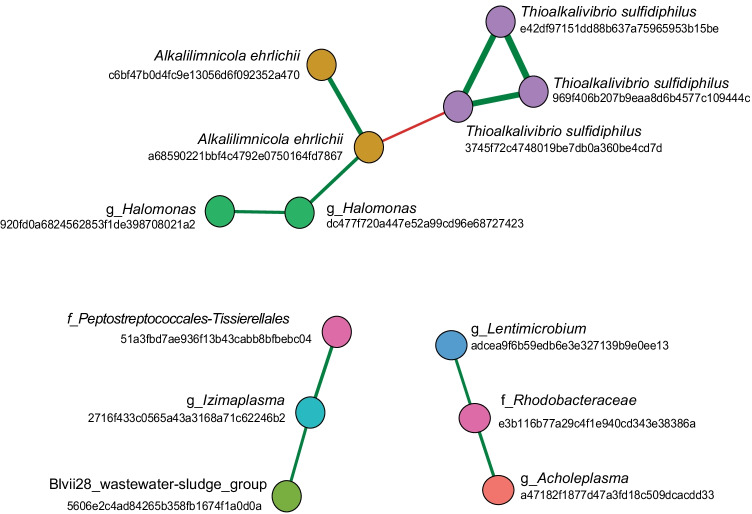
Co-occurrence network of the core community members (ASVs) with a significant Pearson correlation (*p* > 0.05). The positive correlations are shown with green lines and negative correlations with red lines. The thickness of the lines corresponds to the correlation coefficients

To understand the interaction among the members of the core community, a co-occurrence network was created. Figure 5 shows the co-occurrence of the core community members that have significant interactions. The network shows a negative correlation between the *Thioalkalivibrio sulfidiphilus* and *Alkalilimnicola ehrlichii* genera, a positive correlation between *Alkalilimnicola ehrlichii* and *Halomonas sp.* Likewise, *Lentimicrobium*, *Acholeplasma*, and *Rhodobacteracea* were found to have significant positive correlations in their occurrence. This relationship was also found for the Blvii-wastewater sludge group, *Candidatus* “Izimaplasma,” and members of the family *Peptostreptococcales-Tissierellales*.

### Relation between physicochemical parameters and microbial community composition

**Fig. 6:**
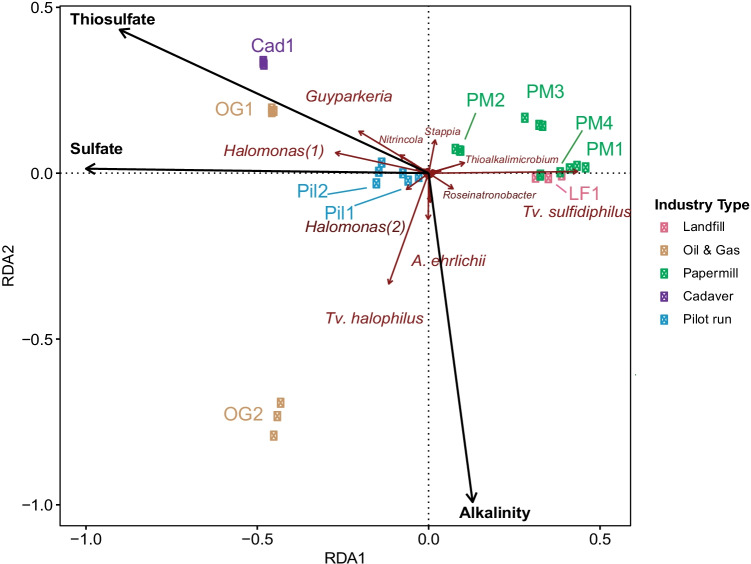
Redundancy analysis (RDA) for 30 samples using 444 ASVs depicted by dark red arrows and 3 statistically significant environmental variables

Redundancy analysis was performed to find a relationship between the microbial community composition and the physicochemical parameters. The parameters that were significant explain the microbial composition with thiosulfate (*R*^2^ = 0.24, *p* < 0.05), sulfate (*R*^2^ = 0.30, *p* < 0.05) and alkalinity (*R*^2^ = 0.46, *p* < 0.05). As shown in Fig. 6, *Guyparkeria*, *Nitrinicola*, and *Halomonas* were associated with the presence of more sulfate and thiosulfate in the samples while the presence of *Tv. halophilus* and *Alkalilimnicola* were more aligned with alkalinity. On the other hand, *Roseinatronbacter*, *Tv. sulfidiphilus*, and *Thiomicrospira* were not associated with any of the tested parameters.

To find the differential abundance of the microbial population with respect to industry type, alkalinity, sulfate, and thiosulfate, the multinomial regression model was fitted with these variables. A fit value of *Q*^2^ = 0.636 was obtained. However, when the model was fitted with only alkalinity, sulfate, and thiosulfate, a fit value of *Q*^2^ = 0.09 was obtained suggesting that these factors alone cannot explain the differential abundances of microbial community members across the samples.

## Discussion

### Physicochemical analyses

In this paper, we have studied several operational and physicochemical parameters associated with BD reactors. Table 1 clearly indicates distinct variability of parameter values in all reactors and served as a good data set for studying the microbial composition across the plants. For the process, a higher rate of accumulation of sulfate and thiosulfate can be seen as an indicator of a decrease in sulfur selectivity (de Rink et al. 2019). For example, the measured levels of thiosulfate levels are indicators for dominating side-product oxidation pathways. Typically, thiosulfate formation cannot be avoided as, when dissolved oxygen and sulfide will chemically react to thiosulfate (de Graaff et al. 2012; Klok et al. 2013). Increased thiosulfate levels indicate that significant amounts of sulfide are chemically oxidized. Furthermore, the absence of thiosulfate indicates that all thiosulfate which is formed is subsequently oxidized to sulfate. While the analyzed concentrations of accumulated ions cannot directly be translated to the rate formation, these results can still be directly related to BD plant performance. Hence, these values are useful to explain the microbial community composition of the plants. For future analyses, the ratio of S_2_O_3_^2–^/SO_4_^2–^ can also help in explaining the presence of SOB that are sulfide specialized and/or more generalists in the BD reactors. Higher S_2_O_3_^2–^/SO_4_^2–^ reflects more chemical oxidation and can also suggest less thiosulfate oxidizing bacteria in the reactor. Additionally, the sampling and physicochemical analyses can be extended to have higher sampling frequencies to visualize the fluctuations in the reactors.

The ORP is considered to play an important role in determining sulfur selectivity. However, recently it has been shown that a constant redox potential does not lead to the highest sulfur selectivity, and a stoichiometry-driven feedforward controlling of oxygen has been suggested to lead to a high sulfur selectivity (Kiragosyan et al. 2020b). Nevertheless, microbial community composition can influence or can be influenced by the ORP and could indirectly affect the sulfur selectivity.

### Microbial diversity analysis

The alpha diversity analyses have indicated that the two pilot plant samples (Pil1 and Pil2) have more diversity as compared to the full-scale BD reactors. This suggests that the addition of an anaerobic bioreactor might provide a niche to specific bacteria, for example, facultative anaerobes (de Rink et al. 2019) that were not present in the full-scale reactors.

The beta diversity analysis of the samples clearly suggests that the microbial communities are significantly affected by the composition and the source of gas of the different industries (Fig. 1). Having an input gas without hydrocarbon contaminants, the biomass of the paper mill (PM1, 2, 3, and 4) and landfill for building material which is more hydrocarbon free unlike usual landfills (LF1), these BD reactors were found to be very similar in their microbial composition while the biomass of the two oil and gas reactors (OG1 and 2) differs from each other in composition as they have distinct contaminants in them. Other studies have shown that the presence of thiols and BTEX have an effect on the microbial community composition in BD reactors (Roman et al., 2016; Kiragosyan et al. 2020a).

The microbial community was found to be less diverse in the BD-reactors that treat feed gas from the same source industry type suggesting the huge impact of the feed gas source. For example, *Tv. sulfidiphilus* was abundant in all paper mill and landfill plants (PM1–4, LF1), while *Thiomicrospira* was more abundant in pilot plant samples (Pil1 and 2). Other process parameters such as the sulfur separation method and the H_2_S/CO_2_ ratio have little effect on the microbial community (Supplementary Fig. S2). However, analysis with more BD reactors could help to get more conclusive results.

### The core microbial community of BD reactors

The microbial diversity analysis of ten BD reactors clearly reveals a shared microbial community composition that is variably abundant across the plants. The 30 ASVs can be defined as the core microbial community because they are shared by all the plants and has no bias of their geographical location, the industrial gas streams they treat, or the differences in the physicochemical parameters. The possible reason for the presence of a core community is the haloalkaline environment in all the BD reactors. The microbiome of soda lakes, a natural haloalkaline environment, also has a core microbiome in lakes located across the continents (Zorz et al. 2019). A metagenome-based study of soda lakes has also highlighted the presence of similar phyla in the soda lakes that were found in BD reactors (Vavourakis et al. 2016). Interestingly, these 30 core community members are not only common but together also comprise a very large proportion of the total microbial diversity of BD reactors (Fig. 2B).

### Taxonomy-based and phylogenetic analyses

Taxonomy-based assignment of the 30 core ASVs (Figs. 2B and 3) indicated that they could be assigned to the *Gammaproteobacteria* (16), *Alphaproteobacteria* (4), *Bacteroidia* (4), *Bacilli* (3), *Clostridia* (1), *Desulfuromondia* (1), and *Chrysiogenetes* (1)*.* It is worth noting that within 30 core ASVs, there are ASVs that were assigned similar phylogenies even though they have different sequences. BLASTn analysis of these sequences has revealed that sequences vary in identity (96 to 100%) and coverage (90 to 100%). Based on this analysis, it is possible that they represent different species of bacteria or different strains of the same species. However, considering the fact that most common SOB such as *T. sulfidiphilus* and *A. ehrlichii* are not very phylogenetically distant (Ahn et al. 2017), we have considered all 30 ASVs as individual bacteria.

**Fig. 7:**
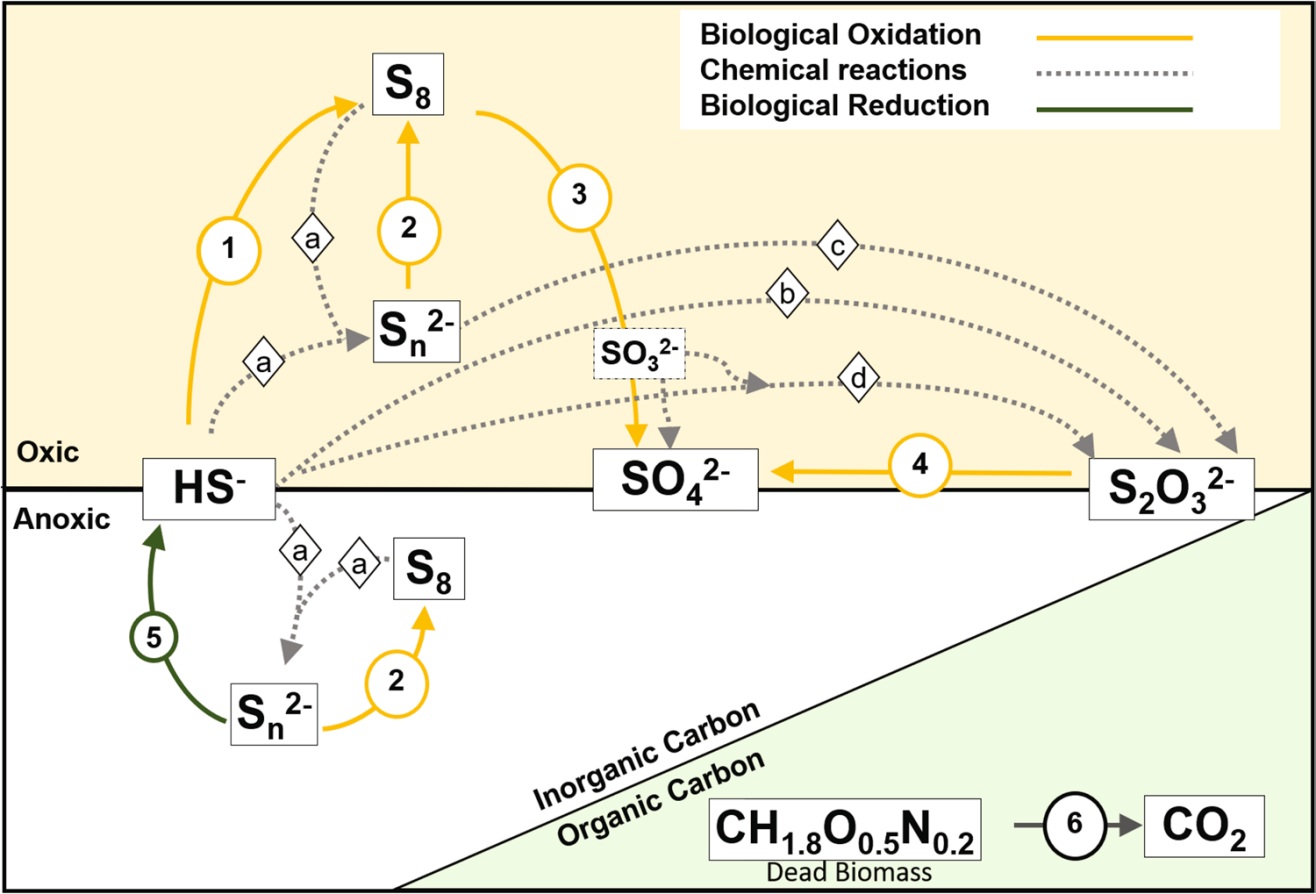
Biological and chemical conversions occurring in the BD reactors. The numbers represent the biological conversions performed by the core community members. The letters denote the chemical reactions. The description of the numbers and letters are specified in Table 2

In general, several known SOBs belong to the class *Gammaproteobacteria* such as species of *Thioalkalivibrio*, *Thiomicrospira, Alkalilimnicola*, *Guyparkeria*, *Thioalkalibacter*, and *Alkalispirillum* (Rijkenberg et al. 2001; Sorokin et al. 2001, 2012; Hoeft et al. 2007; Banciu et al. 2008; Boden 2017)*.* The first four were also part of the core community, indicative of their important role in sulfide oxidization in the BD process (Fig. 7), which was confirmed by the predicted abundance of the sqr and fccAB genes (Fig. 4). The later ones were only abundantly present in a few reactors. In addition, the core community also consisted of other members assigned as *Ectothiorhodospira*. Members of this phototrophic genus can oxidize sulfur compounds. Other genera belonging to a group of 16 ASVs contained *Halomonas*, *Nitrincola*, and *Wenzhouxiangella. Halomonas* are known organotrophs but can also oxidize sulfur compounds incompletely to tetrathionate (Sorokin 2003). Some of the denitrifying species have the potential to oxidize sulfide and thiosulfate anaerobically (Sorokin 2003; Wang and Shao 2021). *Nitrincola* is a facultatively anaerobic, chemo-organotrophic bacterium capable of nitrate reduction but not known for sulfide oxidation. But they contain potential genes for thiosulfate dehydrogenase yielding tetrathionate (Vavourakis et al. 2016; Borsodi et al. 2017). A novel haloalkaliphilic species of the genus *Wenzhouxiangella* sp. has recently been enriched and isolated from Siberian soda lakes using cells of Gram-positive cocci as substrate and was proven to be strongly proteolytic with the ability to feed on microbial biomass (Sorokin et al. 2020). In all, these obligate heterotrophic haloalkaliphiles can be considered a “satellite” population of the autotrophic SOB feeding on their organic products with an ability to marginally contribute to sulfur cycling, such as by the production of tetrathionate.

Members of the class *Alphaproteobacteria* and family *Rhodobacteraceae* such as *Roseinatronbacter* are aerobic sulfur-oxidizing lithoheterotrophs that oxidize inorganic sulfur compounds to sulfate during organotrophic growth (Sorokin et al. 2000; Boldareva et al. 2007; Gorlenko et al. 2010). *Stappia* is well known to be involved in denitrification and CO oxidation (Weber and King 2007). However, it has genes to convert thiosulfate to sulfate (Huang et al. 2015a) and has been found in several sulfide-removing reactors (Huang et al. 2015b; San-Valero et al. 2019). Their role in BD reactors is shown in Fig. 7.

The ASVs assigned to *Bacteroidota* include *Planktosalinus*, *Lentimicobium*, uncultured_Bacteroidetes/Chlorobi, and *Psychroflexus*. So far only one member of *Planktosalinus*, *P. lacus* has been characterized as a strict aerobic heterotroph (Zhong et al. 2016). For *Lentimicrobium, L. saccharophilum* is a strict anaerobe and cannot use any sulfur compounds as electron acceptors (Sun et al. 2016). Uncultured_Bacteroidetes/Chlorobi assigned to the Blvii28_wastewater-sludge_group are members of the core community, and they are frequently detected in sulfide-oxidizing bioreactors (Vannini et al. 2008). However, the specific role of this Blvii28_wastewater-sludge_group is not clear. On the other hand, *Chlorobi*, the green sulfur bacteria, are known to perform sulfide oxidation (Fig. 7) (Visser et al. 1997; Ghosh and Dam 2009). Members of the genus *Psychroflexus* and, in particular, *P. torquis* are very versatile bacteria that use an extensive range of carbon and energy sources, including carbohydrates, amino acids, organic acids, and odd chain length lipid oxidation products (Bowman et al. 1998).

Members of class *Bacilli* namely *Acholeplasma* and *Candidatus* “Izimaplasma” are not known to participate in the sulfur cycle but may play a role in degrading organic matter present in the BD reactors (Skennerton et al. 2016; de Rink et al. 2019; Zorz et al. 2019). Member of class *Clostridia*, order *Peptostreptococcales-Tissierellales* are also part of the core community. Some of these members are strictly anaerobic and can reduce elemental sulfur (Fig. 7) (Takai et al. 2001), and some are haloalkaliphilic acetogens that cannot reduce sulfate (Pikuta et al. 2003). Lastly, members of the class *Desulfuromondia* and *Chrysiogenetes*, namely, *Desulfuromosa* and *Desulfurisprillum* are anaerobic organotrophs and can use elemental sulfur as an electron acceptor (Fig. 7) (Liesack and Finster 1994; Sorokin et al. 2007).

Figure 7 summarizes the possible biological conversions that involve one or more core members in the BD reactors, and Table 2 lists the bacteria associated with these conversions. The figure suggests that the core community consisted of bacteria with diverse functions. While for some SOB, the role can be clearly associated with several oxidation steps of sulfide, there are also thiosulfate oxidizers and sulfur reducers present but no sulfate reducers. There are also several members which are heterotrophs and whose potential role in sulfur conversions is not known; however, metagenomic analyses of natural and other sulfide-related reactors have also listed some of these bacteria. This could mean that these bacteria use the organic matter from decomposed bacterial cells and their role in the reactor is an opportunist scavenger. Tetrathionate could also be formed by heterotrophic bacteria from thiosulfate oxidation (Sorokin 2003). Tetrathionate is instable at high pH (>10) and is chemically decomposed to thiosulfate, trithionate, and pentathionate (Varga and Horváth 2007). It can also react with sulfide to form sulfur and thiosulfate. Furthermore, some bacteria can biologically convert tetrathionate to sulfur, sulfate, and thiosulfate (Sorokin 2003). Due to the above-mentioned conversions, tetrathionate is less likely to be available as an intermediate in the BD reactors and therefore contributes little, if at all, to the sulfur conversions. This is also shown by the low abundance of the genes involved in tetrathionate conversions (Table S2).

**Table 2:** Biological and chemical conversions occurring in the BD reactors with the potential role of each core community member

Biological conversions	Reaction	Core ASVs
***Sulfide oxidation***		
Complete sulfide oxidation HS^-^ → SO_4_^2–^	1, 3	*Thioalkalivibrio, Guyparkeria, Thioalkalispira (*formerly *Thioalkalimicrobium), Ectothiorhodospiraceae, Rhodobacteraceae, Roseinatrobacter*
Partial sulfide oxidation HS^-^ → SO_8_	1	*Alkalilimnicola*
***Polysulfide oxidation***		
Complete polysulfide oxidation HS_n_^2–^ → SO_4_^2-^	2,3	*Thioalkalivibrio, Guyparkeria, Thioalkalispira (*formerly *Thioalkalimicrobium), Ectothiorhodospiraceae, Rhodobacteraceae, Roseinatrobacter*
Partial polysulfide oxidation S_n_^2–^→ S_8_	2	*Alkalilimnicola*
***Thiosulfate oxidation***		
Thiosulfate oxidation to sulfate S_2_O_3_^2–^ → SO_4_^2–^	4	*Thioalkalivibrio, Guyparkeria, Thioalkalispira (*formerly *Thioalkalimicrobium), Ectothiorhodospiraceae, Rhodobacteraceae, Roseinatrobacter*
***Sulfur reduction***		
Sulfur reduction S_8_→ HS^–^	5	*Desulfuromusa, Desulfurispirillum*
***Heterotrophic growth***		
Organic carbon assimilation by heterotrophs CH_1.8_O_0.5_N_0.2_→ CO_2_	6	*Nitrincola, Halomonas, Wenzhouxiangella, Planktosalinus, Psychroflexus, Lentimicrobium, Blvii28 wastewater sludge group, Acholeplasma, Izimaplasma, Peptostreptococcales-Tissierellales*
**Chemical conversions**		
Polysulfide formation HS^–^ + S_8_→ S_n_^2–^	a	
Sulfide oxidation HS^–^ → S_2_O_3_^2–^	b	
Polysulfide oxidation S_x_^2–^ → S_2_O_3_^2–^	c	
Sulfite and sulfide reaction 2HS^–^ + 4HSO^–^ → 3S_2_O_3_^2–^ + 3H_2_O	d	

In addition to the biological reactions, Figure 7 also shows the possible chemical conversions that play an important role in generating alternative substrates for SOB, like polysulfides and thiosulfate.

The core ASVs shape the major fraction of the total microbial community, but there are other species that were abundant only in some of the BD reactors (Fig. 2B). *Alkalisprillum* was abundant (14.3%) in Papermill_02, *Vibrio* (16.2%) in Papermill_03 and *Nitrincola* (13.8%) in Cadaver_01. *Alkalisprillum* is a close relative of *Alkalilimnicola* and is known to oxidize sulfide/polysulfide to elemental sulfur aerobically (Sorokin et al. 2006). Members of genera *Vibrio* are widely found in marine and haloalkaline environments (Simidu and Tsukamoto 1985; Mwirichia et al. 2010). Studies have predicted that members of the family *Vibrionacea* have the potential to oxidize thiosulfate to tetrathionate with heterotrophic growth (Sorokin 2003).

### Prediction of the functional potential of the core community

Tax4Fun2 predicted the overall high abundance of genes involved in the oxidation of sulfide to sulfur (sqr; fccAB; Fig. 4). However, sqr and fccAB were variably present in different reactors suggesting that the mechanism of sulfide oxidation might differ among the different industry reactors. The possibility of variable mechanisms was also shown in the previous studies on the sludge from full-scale plants (Kiragosyan et al. 2019). Additionally, paper mill (PM1–4) and landfill-treating reactors (LF1) have a similar functional profile. These plants were dominated by *Thioalkalivibrio sulfidiphilus* which has fccAB genes and a sox gene system but lacks the sqr gene. Contrarily, the rendering waste treating reactor (Cad1) has a very different functional profile than others with a high abundance of sqr genes and less of fcc and sox genes. The pilot runs studied in this research have an abundance of both fcc and sqr. These results differ from the previous research where sqr was suggested to be most abundant (de Rink et al. 2019). All these predictions indicate the differences in the potential mechanisms of sulfide oxidation among the reactors. However, to make a definite conclusion, more information on the sulfide loads and sulfur selectivity along with gene expression results is needed.

### Co-occurrence analyses

Co-occurrence analysis of the core community has revealed an antagonistic relationship between *Tv. sulfidiphilus* and *A. ehrlichii* (Fig. 5). Recent studies with the modified BD line-up have also indicated these dynamics in the reactors (de Rink et al. 2019). Being both a facultative autotroph and facultative anaerobe, *A. ehrlichii* is more versatile than *T. sulfidiphilus*, which is a strict autotroph. *A. ehrlichii* was found to co-occur with *Halomonas* sp*.*, indicating the presence of organic compounds in the reactor. The analysis also shows that there are two sets of heterotrophic bacteria from different phyla co-occurring in the BD reactors.

### Relation between physicochemical parameters and microbial community composition

Though the biological process of full-scale BD reactors is the same, there are several operational and design parameters that make them different. RDA analysis indicated that the concentration of thiosulfate, sulfate, and alkalinity in the BD reactors is related to the presence of certain bacteria. Figure 7 highlights that members of the core community have a different potential to oxidize sulfur compounds. The complete/incomplete biological sulfide oxidation can thus lead to the formation of sulfate/sulfur. Moreover, different SOBs, such as *Thioalkalivibrio* and *Thiomicrospira* have different growth strategies (Sorokin et al. 2003).

From the results of RDA analysis (Fig. 6), it is evident that *Guyparkeria* and *Halomonas* were found to be highly correlated with the increasing concentration of thiosulfate and sulfate in the BD process. *Guyparkeria* can oxidize several reduced sulfur compounds and oxidize them to sulfate (Sievert et al. 2000) and can decrease the alkalinity of a neutral environment They are also capable to drive a neutral environment to less alkalinity (Whaley-Martin et al. 2019). This can be explained by an increase in H^+^ ions concurrently formed with sulfate production. On the other hand, *Alkalilimnicola* was more correlated with high alkalinity.

As mentioned before, in a BD reactor a high rate of thiosulfate and sulfate formation together with their accumulated concentration indicates a sub-optimal process performance. Nevertheless, considering only the accumulated concentration, *Guyparkeria* and *Halomonas* can be regarded as indicator organisms of high sulfate and thiosulfate accumulation in the process. High thiosulfate and sulfate formation directly corresponds to a decreased alkalinity and concomitantly an increase in the caustic demand, thereby decreasing the performance of the BD process and increasing costs.

To conclude, microbial community analysis of ten full-scale BD reactors treating gas streams from different industries demonstrated the presence of a core microbiome consisting of 30 ASVs with *Thioalkalivibrio* *sulfidiphilus* as the most abundant community member. The diversity is largely affected by the source and composition of the feed gas. Members of the genera *Guyperkaria* and *Halomonas* are related to an increase in sulfate and thiosulfate levels and as such can be regarded as indicator organisms for a suboptimal performance of the BD processes.

In this study, only a few physicochemical parameters such as pH, salinity, alkalinity, conductivity, thiosulfate, sulfate concentrations have been considered. In the future, it is recommended to include parameters such as ORP, sulfide load, airflow rate, and sulfur selectivity while performing microbial composition analysis for these reactors.

## Supplementary Information

Below is the link to the electronic supplementary material.

Supplementary file1 (PDF 443 KB)

## Data Availability

All the data and material has been shared as part of the manuscript. The amplicon sequences of this study can be found in the Sequence Read Archive under PRJEB43104.
