# Supplementary material for: Comparative analysis of microbial communities from different full-scale haloalkaline biodesulfurization systems
Source: Appl Microbiol Biotechnol. 2022 Feb 11;106(4):1759–76. doi: 10.1007/s00253-022-11771-y (PMC8882115; doi:10.1007/s00253-022-11771-y)
Supplement: Supplementary file 1 — Supplementary file1 (PDF 443 KB) [file 253_2022_11771_MOESM1_ESM.pdf]

Applied Microbiology and Biotechnology

SUPPLEMENTARY INFORMATION

Comparative analysis of microbial communities from different full-scale haloalkaline biodesulfurization systems

Suyash Gupta[1,2], Caroline M. Plugge[1,3], Johannes B. M. Klok[1,4], Gerard Muyzer[2]

[1]Wetsus, European Centre of Excellence for Sustainable Water Technology, Leeuwarden, The Netherlands

[2]Microbial Systems Ecology, Department of Freshwater and Marine Ecology, Institute for Biodiversity and Ecosystem Dynamics, University of Amsterdam, Amsterdam, The Netherlands

[3]Laboratory of Microbiology, Wageningen University & Research, Wageningen, The Netherlands

[4]Paqell B.V., Utrecht, The Netherlands

Corresponding author: g.muijzer@uva.nl

Table S1. Number of sequence reads for the samples collected in 2018.

Sample Id	Number of reads
**Cad1a**	48400
**Cad1b**	60272
**Cad1c**	73228
**LF1a**	56050
**LF1b**	60243
**LF1c**	63710
**OG1a**	62969
**OG1b**	56007
**OG1c**	55224
**OG2a**	41722
**OG2b**	43094
**OG2c**	59317
**Pil1a**	63273
**Pil1b**	56281
**Pil1c**	56661
**Pil2a**	67918
**Pil2b**	59129
**Pil2c**	55390
**PM1a**	41137
**PM1b**	47904
**PM1c**	39743
**PM2a**	60763
**PM2b**	60444
**PM2c**	50849
**PM3a**	68109
**PM3b**	61711
**PM3c**	54626
**PM4a**	54829
**PM4b**	51598
**PM4c**	33342

Table S2 The relative abundance (x10^{-5}) of functional genes obtained by Tax4Fun2

KO	Description	Cad1	LF1	OG1	OG2	Pil1	Pil2	PM1	PM2	PM3	PM4
K17218	sulfide:quinone oxidoreductase (sqr)	81	12	9	10	25	65	16	24	22	1
K17229	cytochrome subunit of sulfide dehydrogenase (fccA)	47	87	39	61	53	95	83	70	83	99
K17230	sulfide dehydrogenase [flavocytochrome c] flavoprotein chain (fccB)	55	92	42	48	67	108	85	75	85	99
K08352	thiosulfate reductase / polysulfide reductase chain A (psrA)	1	0	4	26	21	32	0	1	1	3
K17222	sulfur-oxidizing protein SoxA	35	120	44	30	54	41	112	97	110	132
K17224	sulfur-oxidizing protein SoxB	29	34	14	49	24	31	30	29	35	34
K17225	sulfane dehydrogenase subunit SoxC	29	38	13	26	31	33	31	31	33	33
K17223	sulfur-oxidizing protein SoxX	35	120	44	54	55	42	112	97	113	132
K17226	sulfur-oxidizing protein SoxY	34	63	27	55	54	66	57	52	59	67
K17227	sulfur-oxidizing protein SoxZ	34	63	27	46	47	56	57	52	59	66
K03388	heterodisulfide reductase subunit A2 (hdrA)	3	29	13	34	18	15	28	24	29	36
K03389	heterodisulfide reductase subunit B2 (hdrB)	5	58	21	50	21	8	55	46	57	66
K03390	heterodisulfide reductase subunit C2 (hdrC)]	5	58	21	50	21	8	55	46	57	66
K21307	sulfite dehydrogenase (quinone) subunit SoeA	4	34	12	66	32	28	30	26	31	34
K21308	sulfite dehydrogenase (quinone) subunit SoeB	3	34	11	58	25	17	30	26	31	34
K21309	sulfite dehydrogenase (quinone) subunit SoeC	3	34	11	58	25	17	30	26	31	34
K08357	tetrathionate reductase subunit A (ttrA)	0	0	1	0	0	0	0	0	0	1
K08358	tetrathionate reductase subunit B (ttrB)	0	0	4	9	7	11	0	1	1	4

K08359	tetrathionate reductase subunit C (ttrC)	0	0	1	0	0	0	0	0	0	1
K11180	dissimilatory sulfite reductase alpha subunit A (dsr A)	2	29	11	10	17	14	28	23	26	33
K11181	dissimilatory sulfite reductase alpha subunit B (dsr B)	2	29	11	10	17	14	28	23	26	33
K00958	sulfate adenylyltransferase (sat)	2	29	11	25	18	5	28	25	28	33
K00956	sulfate adenylyltransferase subunit 1 (sat)	3	0	37	16	7	12	0	1	1	2
K00957	sulfate adenylyltransferase subunit 2 (sat)	20	6	38	25	15	25	8	10	9	2
K19713	thiosulfate dehydrogenase(tsdA)	17	30	23	68	25	26	28	27	36	34
K03381	catechol 1,2-dioxygenase	8	5	6	4	11	2	9	4	3	0
K07104	catechol 2,3-dioxygenase	1	6	7	5	1	0	1	1	6	0

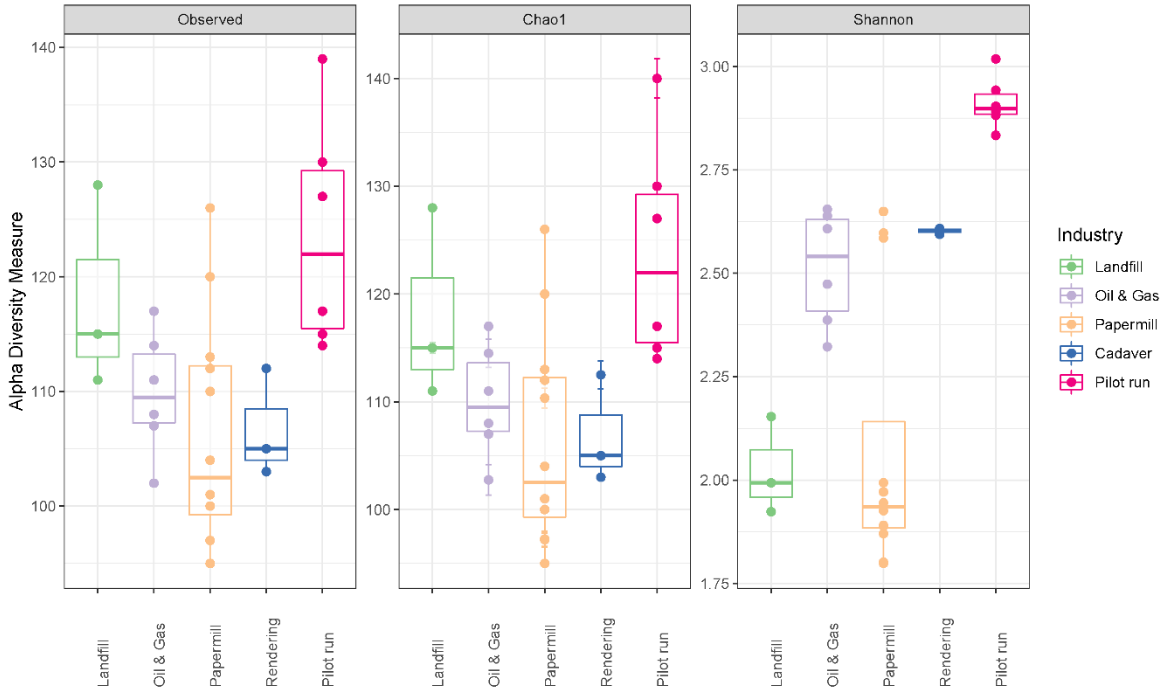

Fig S1. Alpha diversity of microbial communities from biomass samples collected in of 2018. The results are grouped by industry type and based on Observed OTUs, Chao1 and Shannon alpha diversity indices.

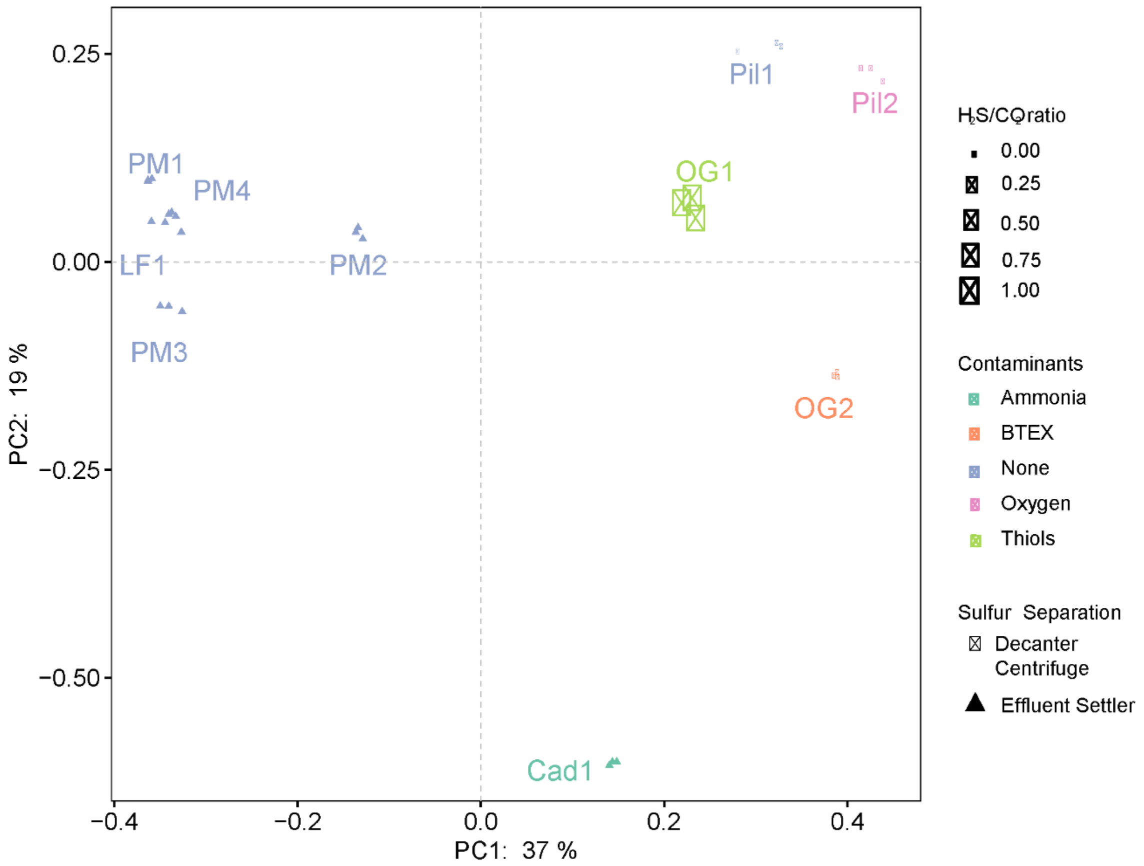

Fig S2. PcoA plot of 2018 samples (three replicates) showing the differences in microbial community composition. These differences might be caused by contaminants in the feed gas, the sulfur separation method and the feed gas H_2S/CO_2 ratio.
